# Pathophysiological Changes in Erythrocytes Contributing to Complications of Inflammation and Coagulation in COVID-19

**DOI:** 10.3389/fphys.2022.899629

**Published:** 2022-06-15

**Authors:** Prashilla Soma, Janette Bester

**Affiliations:** ^1^ Department of Anatomy, University of Pretoria, Pretoria, South Africa; ^2^ Department of Physiology, University of Pretoria, Pretoria, South Africa

**Keywords:** coagulopathy, cytokines, precision medicine, COVID-19, erythrocytes

## Abstract

Higher thrombotic burden in the acute phase of COVID-19 relies on a complex interplay between pro-inflammatory cytokine/chemokine release, increased endothelial dysfunction/damage, and potential sepsis-induced coagulopathy development in severe cases, all promoting coagulation activation. Plasma levels of cytokines and chemokines are known to be increased in COVID-19 however, are much higher in severe infections. Increased levels of IL-1β, IL-6, and IL-8 are known to play an important role in both acute and chronic inflammation, resulting in pathological clotting. However, little has been published on the effects of these interleukins on red blood cells (RBCs). Evidence shows that cytokines have a negative effect on the RBCs ultrastructure and introduce signs of eryptosis. Eryptosis can be described as a form of suicidal death of RBCs characterized by distinct findings of cell shrinkage, membrane blebbing, activation of proteases, and phosphatidylserine exposure at the outer membrane leaflet. Red blood cells from COVID-19 patients had increased levels of glycolytic intermediates, accompanied by oxidation and fragmentation of ankyrin, spectrin beta, and the N-terminal cytosolic domain of band 3 (AE1). Significantly altered lipid metabolism was also observed, in particular, short- and medium-chain saturated fatty acids, acyl-carnitines, and sphingolipids. Emerging research suggests that RBCs may contribute to a precision medicine approach to sepsis and have diagnostic value in monitoring complement dysregulation in COVID-19-sepsis and non-COVID sepsis as research indicates that complement activation products and viral antigens are present on RBCs in patients with COVID-19.

## Introduction

Since 1856, the aetiology and risk of thrombosis have been assessed using Virchow’s triad. This triad consists of stasis, vessel damage, and hypercoagulability (David et al., 2009). Our understanding of thrombosis has since then evolved and a modern interpretation of this triad includes: hemodynamic disruption, intrinsic hypercoagulability, and endothelial damage or dysfunction as the three broad categories that lead to thrombosis (Monie and Deloughery, 2017). The importance of this triad in disease-associated complications lies in combining the inflammatory and coagulation pathways in the genesis of clotting (Ahmed et al., 2020).

COVID-19 has been regarded as an infective-inflammatory disease and it has become increasingly clear that high levels of pro-inflammatory mediators play a major role in the clinical deterioration in patients with severe disease. Recent evidence indicates a tendency for thrombosis in COVID-19 patients (Ahmed et al., 2020). Given the ongoing global pandemic, there is an urgent need to understand the rate of bleeding and thrombotic manifestations associated with COVID-19 coagulopathy (Grobler et al., 2020). Even though most of the patients infected with COVID-19 experience only mild symptoms there is a considerable percentage of patients that deteriorate significantly, causing multiple organ failures that result in death (Martha et al., 2021). Higher thrombotic burden in the acute phase of COVID-19 relies on a complex interplay between pro-inflammatory cytokine/chemokine release, increased endothelial dysfunction/damage, and potential sepsis-induced coagulopathy development in severe cases, all promoting coagulation activation (Marchandot et al., 2020). A hypercoagulable state in patients with severe COVID-19 has emerged as multiple changes in circulating prothrombic factors have been detected such as elevated factor VIII, increased fibrinogen, circulating prothrombic microparticles, neutrophil extracellular traps and signs of hyperviscosity (Maier et al., 2020; Panigada et al., 2020; Ranucci et al., 2020). Multiple studies performed on subjects with COVID-19 have shown laboratory results in severe disease that included: elevated lactate dehydrogenase (LDH), serum ferritin, D-dimer, and creatine kinase (Ponti et al., 2020; Ragab et al., 2020; Tian et al., 2020). A meta-analysis was done by Henry et al., totalling a number of 2984 COVID-19 patients also reported laboratory data with a comparison between those with severe and non-severe disease and provided a list of laboratory results in patients with severe or fatal COVID-19. Amongst these results, they showed that patients with severe disease had increased levels of ESR, IL-6, IL-8, CRP, serum ferritin, and LDH (Henry et al., 2020). It is suggested that the increased cytokines contributed to the cytokine storm seen in patients with severe disease (Ragab et al., 2020; Hu et al., 2021). The cytokine storm is attributed to the action of the pro-inflammatory cytokines such as IL-1, IL-6, IL-18, and TNFα (Ponti et al., 2020; Ragab et al., 2020; Zeng et al., 2020; Hu et al., 2021).

### Mechanism of Activating the Cytokine Storm

Severe acute respiratory syndrome coronavirus 2 (SARS-CoV-2) infection of type I and II pneumocytes causes virus-related epithelial pyroptosis and activation of macrophages in the pulmonary alveoli (Argañaraz et al., 2020; Pearce et al., 2020). The acute-phase response cytokines, IL-6 and TNF-α, are suggested to be the major ‘culprits’ in the pathogenesis of COVID-19 hyper-inflammation (Panigrahy et al., 2020). It has been suggested that IL-6 promotes a macrophage activation syndrome (MAS), triggering mass production of pro-inflammatory cytokines and inducing migration of neutrophils and fibroblasts into the pulmonary epithelium (Pearce et al., 2020). Activated macrophages are the main source of pro-inflammatory cytokines such as IL-1β, IL-6, IFN-γ, IL-8, and TNF-α (Argañaraz et al., 2020). The cytokine IL-6 induces endothelial activation and inflammatory cell migration. There is an increase in tissue factor (TF) secretion and upregulation of the coagulation cascade leading to immune-mediated thrombosis (Pearce et al., 2020). Endothelial activation stimulates inflammatory cross-talk contributing to a second cytokine wave.

Increased levels of IL-1β, IL-6, and IL-8 are known to play an important role in both acute and chronic inflammation, with resulting pathological clotting (Bester and Pretorius, 2016). However, little has been published on the effects of these interleukins on RBCs. In 2016, Bester et al., showed that increased levels of IL-8 had a negative effect on the RBCs ultrastructure and introduced signs of eryptosis (Bester and Pretorius, 2016). Red blood cells are extremely vulnerable cells, particularly during inflammation, whether it is acute or chronic (Pretorius et al., 2016). A study by Darbonne et al., established that there were surface binding proteins for IL-8 on RBCs and that these cells act as a general intravascular sink for soluble chemotaxins for modulating inflammation (Darbonne et al., 1991; Karsten et al., 2018). In a review Karsten and Herbert described the Duffy antigen receptor for chemokines (DARC) on RBCs as a chemokine scavenger which is able remove or release cytokines as needed (Fukuma et al., 2003; Karsten and Herbert, 2020). Here they also added that DARC is activated by clotting and the release of chemokines occurs in response to clotting which links DARC as a key factor in inflammation (Fukuma et al., 2003; Karsten and Herbert, 2020). Therefore it can be said that RBCs may also be in immuno-stimulatory and may contribute to the cytokine storm (Karsten and Herbert, 2020).

### Effect of COVID-19 on the Red Blood Cells

The morphology of the RBC in patients presenting with COVD-19 related with anemia, was observed by Berzuini et al., where peripheral blood smears were analysed. Their unsuspected findings showed several RBCs shape abnormalities with a high frequency of stomatocytes and knizocytes. Other features observed were that of polychromasia, basophilic stippling, rouleaux formations, and autoagglutination (Berzuini et al., 2021).

**FIGURE 1:**
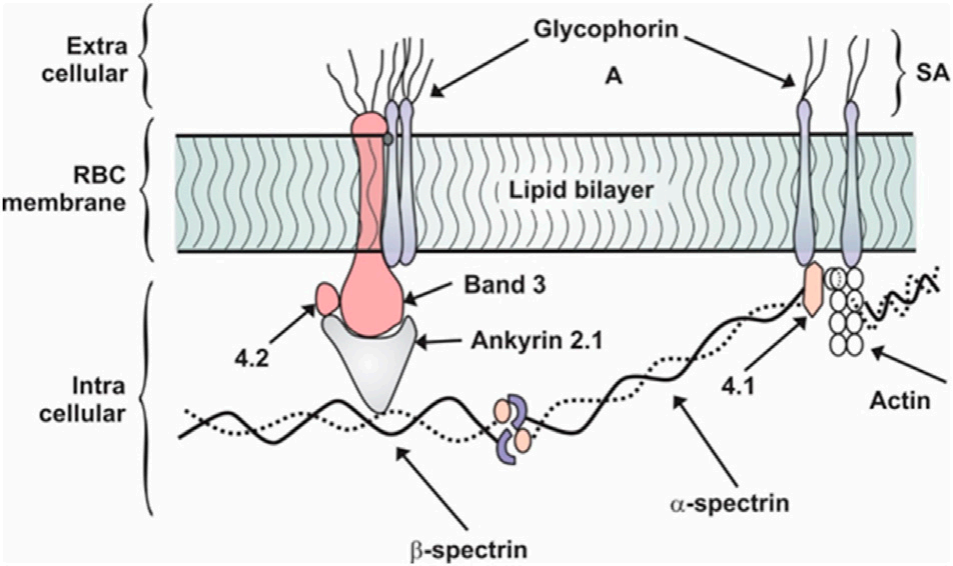
Schematic representation of RBC membrane showing protein orientation. Adapted from (Desouky 2009). SA, sialic acid.

Red blood cells have a complex membrane structure as shown in Figure 1 that is continuously exposed to inflammatory molecule insults, inducing a programmed cell death specific to RBCs, known as eryptosis. By definition, eryptosis is a fundamental cellular death process of RBCs similar to apoptosis of nucleated cells (Qadri et al., 2017). Eryptosis is characterized by RBC shrinkage, cell membrane blebbing, and cell membrane scrambling with phosphatidylserine (PS) translocation to the RBC surface. In the pilot study exploring the link between SARS-CoV-2 and RBC physiology, findings suggests that the virus induces increase of IgG’s on the RBC surface, RBC oxidative stress, with accompanying increase in intracellular Ca^2+^ as well to increase RBC’s fragility to mechanical stress (Bouchla et al., 2022). In the setting of sepsis, increased RBC intracellular reactive oxygen species have been known to result in decreased RBC deformability, changes similar to seen with eryptosis (Pretorius 2018). Another adverse effect of the increased intracellular Ca^2+^ is to cause the translocation of membrane PS to the outer space of the RBC membrane. This action stimulates thrombosis through the formation of microparticles and the prothrombinase complex assembly (Bouchla et al., 2022).

Research performed by Piagnerelli et al., showed that in COVID-19 patients with acute respiratory distress syndrome, especially non-survivors, had an altered RBC shape as assessed by flow cytometry, but no change in RBC deformability was found. However, RBC deformability in those patients with bacterial sepsis, was significantly altered (Piagnerelli et al., 2022).

Proteins found on the RBC membrane are classified into two groups: integral and peripheral as shown in Figure 1. Integral proteins are the glycophorin and band 3 which are tightly bound to the membrane through hydrophobic interactions lipids in the bilayer. A filamentous network of proteins is anchored to the bilayer by the integral proteins. This network is made up of three principal components: spectrin, actin, and protein 4.1, details of which are shown in Figure 1 (Thangaraju et al., 2020). The protein band 4.1 stabilizes the attachment of spectrin and actin. Holding the cytoskeleton and bilayer together is the protein ankyrin which anchors spectrin to band 3 and via band 4.1 to glycophorin as clearly defined in Figure 1. The non-linear spring action of spectrin is revealed under the action of an atomic force microscopy tip as the individual spectrin chain is stretched (Shin et al., 2007). These membrane proteins provide specific functions. The most abundant trans-membrane protein is band 3 and it is responsible for anion exchange at the level of the plasma membrane (Azouzi et al., 2018). A red blood cell contains 1.2 million band 3 and 0.5 million glycophorin molecules (Johansson and Falk, 2021). Due to the serious clinical complications such as hypoxia and acute respiratory distress syndrome observed in COVID-19, there has been a re-evaluation of RBC-virus interactions. Thomas et al., showed that RBCs from 29 COVID-19 patients had increased levels of glycolytic intermediates, accompanied by oxidation and fragmentation of several proteins (ankyrin, spectrin beta, and the cytosolic domain of band 3 (Thomas et al., 2020). Of particular interest was the observation of oxidation of the N-terminal of band 3. In addition, analysis of clinical hematological parameters such as red blood cell count, hematocrit and mean corpuscular hemoglobin concentration remained relatively normal in COVID-19 infected patients (Thomas et al., 2020). However, mortality risk in COVID-19 appears to be associated with an increased level of red blood cell distribution width (RDW). In a meta-analysis of COVID-19 patients, higher levels of RDW were associated with more severe disease (Zinellu and Mangoni, 2021).

A recent study by Cosic et al., 2020 provides new insight into the pathophysiology of COVID-19 and the RBC. Their findings are based on outcomes measured using a biophysical model, in which band 3 was suggested to be the point of attachment for SARS-CoV-2 on the RBC surface stating that there might be a likely interaction between the RBC band 3 surface protein and the S1 spike protein in the SARS-CoV-s virus (Cosic et al., 2020). This would corroborate findings by Thomas et al., 2020 where the oxidation of band 3 can be attributed to the direct binding of the virus (Thomas et al., 2020). The degradation of band 3 will adversely affect the physiology of the RBC. Disruption of the normal physiology will lead to impaired function of the RBC, a significant one being the delivery of oxygen (Gallagher, 2016). Hypoxia is a common finding in patients with severe COVID-19 disease. The postulation that the effect of the virus on the RBC is due to binding to surface receptors, is likely. However, it is unlikely that the source of attachment of the SARS-CoV-2 is *via* the angiotensin-converting enzyme 2 (ACE2) receptor on the RBC (Cosic et al., 2020). The absence of the ACE2 membrane protein on the RBC, has been confirmed by the erythrocyte proteome database (https://rbcc.hegelab.org) (Hegedüs T et al., 2015).

### Role of Eryptosis in Thrombosis

A few important functions of eryptosis include its preventative role in limiting premature hemolysis of damaged RBCs, its defensive function in malaria-infested RBCs where eryptosis accelerates the clearance of RBCs infected by plasmodium, and in conditions such as polycythemia vera, eryptosis facilitates the removal of excessive RBCs in circulation (Qadri et al., 2017). Emerging evidence shows that eryptotic RBCs exhibit an increased tendency of adhering to endothelial cells as well as platelets. This behaviour theoretically, thus increases the propensity to halt microcirculation as well as contribute to thrombosis due to their procoagulant characteristic features (Borst et al., 2011).

There is also mounting evidence that the biochemical and biophysical properties of RBCs may actively contribute to the pathophysiology of hypercoagulability in an array of clinical conditions (Pretorius and Lipinski, 2013; Pretorius et al., 2016; Emmerson et al., 2018). Some common conditions and diseases associated with enhanced eryptosis include dehydration, hypoxia, iron deficiency anemia, metabolic syndrome, diabetes mellitus, heart failure, and sepsis (Aleman et al., 2014).

The signal that initiates eryptosis is the exposure of PS on the outer membrane leaflet of RBCs (Guo et al., 2018). Upon activation or injury, cells may expose PS on their external surfaces, which facilitates the movement of coagulation proteins onto their membrane surfaces, thereby promoting the formation of a “thrombogenic membrane” which now interacts in the coagulation cascade terminating in the production of thrombin (Lind, 2021). The exposed PS on the outer membrane allows it to act as a catalytic surface for factor Xa and thrombin formation (Guo et al., 2018). Research suggests that RBCs enhance functional coagulation properties and platelet aggregation (Brown et al., 2014). This is in striking contrast to their traditional physiological role of oxygen transport (Brown et al., 2014). In fact, RBCs have been localized in coronary atherosclerotic plaques (Virmani and Roberts, 1983). During RBC aggregation, fibrinogen is believed to serve as a bridging molecule (Weisel and Litvinov, 2019). Research performed on SARS-Cov2-related complications relying on blood foaming, hypothesized that RBC membrane injury during COVID-19 caused by the binding of inflammatory molecules results in serious biophysical events such as bubble nucleation or foaming (Denis 2020). Decompression illness which comprises both arterial gas embolism and decompression sickness is caused by bubbles in blood or tissue during or after a reduction in environmental pressure (decompression). The bubbles are of clinical significance as they can produce mechanical, embolic and biochemical effects. Adverse effects such as capillary leak, extravasation of plasma and haemoconcentration can occur when intravascular bubbles damage the endothelial (Vann et al., 2010). The paper by Denis, postulates that high levels of Angiotensin II, similar to parameters of body temperature and PCO_2_ affects the dissociation curve of hemoglobin and shifts it to the right during RBC transit in the lungs, providing an overload in free oxygen and would involve oxygen supersaturation culminating in bubble nucleation. The end result of foaming would thus worsen the extensive endothelial dysfunction, worsen the gas exchange, trigger the coagulation process, the inflammatory process and the complement pathway, as occurs in decompression illness (Denis, 2020). Decompression illness symptoms includes substernal pain, cough, dyspnoea, progressing to pulmonary oedema, respiratory failure, right ventricular dysfunction and cardiovascular collapse (Vann et al., 2010).

Furthermore, fibrinogen influences RBC function when it directly binds to RBCs by increasing circulating biomarkers by binding to endothelial cells. The presence of an inflammatory biomarker in circulation is associated with reactive oxygen species production, with the consequence of causing eryptosis and pathological deformability in RBCs (Grobler et al., 2020). The study by Venter et al., 2020 highlighted the microscopic changes observed in platelets and RBCs in subjects with COVID-19 and concluded that structural pathologic findings in both platelets and RBCs are key role players in the vascular changes seen in complications related to COVID-19 (Venter et al., 2020).

### COVID-19 Associated Endothelial Dysfunction

Failure by the endothelium layer to perform its functions results in endothelial dysfunction which is defined as a decrease in the bioavailability of vasodilator substances, especially nitric oxide (NO), and an increase in vasoconstrictor substances. The endothelial cells themselves produce NO oxide which is the most important vasodilator substance (Fodor et al., 2021). The significant role of the endothelium in homeostasis is highlighted through reviewing its functions, which includes: 1) modulation of vascular permeability, 2) modulation of vasomotor tone, 3) modulation of coagulation homeostasis, 4) regulation of inflammation and immunity, 5) regulation of cell growth, and 6) oxidation of low-density lipoprotein (LDL) cholesterol (Esper et al., 2006).

Emerging research show that COVID-19 is an endothelial disease (Libby and Lüscher, 2020) with endotheliopathy being directly implicated in the array of adverse effects such as inflammation, cytokine storm, oxidative stress, and coagulopathy (Amraei and Rahimi, 2020). The invasion of SARS-CoV-2 *via* the endothelial cells is facilitated by the high expression level of ACE2 and transmembrane protease serine 2. Another effect of infection with SARS-CoV-2 includes the release of inflammatory cytokines which induce the adhesive property of endothelial cells and in turn promote the infiltration of neutrophils. This subsequently produces reactive oxygen species and neutrophil extracellular traps, eventually causing the injury of endothelial cells. The coagulation process is initiated by activated endothelial cells once they are infected with SARS-CoV-2, leading to platelet binding, fibrin formation, and the clotting of red blood cells, resulting in the adverse effects of systemic thrombosis (Perico et al., 2021).

**FIGURE 2:**
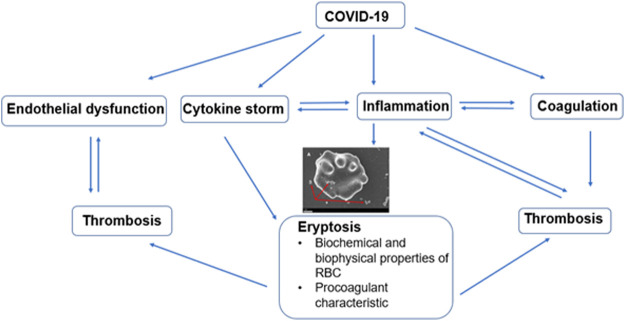
Diagram showing interplay between eryptosis and complications such as endothelial dysfunction, cytokine storm, inflammation, and coagulation. All of which contribute to the increased thrombotic burden observed in COVID-19 disease.

One of the key factors implicated in the pathophysiology of thrombotic complications observed in COVID-19 amongst which includes myocardial infarction and stroke is endothelial dysfunction. In addition, the precise mechanism for the endothelium dysfunction is divided between the direct endothelial cell viral infection or a consequence of the inflammation induced by the virus (Fodor et al., 2021). The interplay between eryptosis and the complications of COVID-19 is illustrated in Figure 2. It can be said that endothelial dysfunction plays a key role.

The post-mortem study examining the lungs of patients who had died from SARS-CoV-2, performed by Ackermann et al., confirmed three distinctive angiocentric features of COVID-19 (Ackermann et al., 2020). The first feature observed was that of a severe grade of endothelial injury (disruption of intercellular junctions, cell swelling, and a loss of contact with the basal membrane of the endothelial cells) associated with intracellular SARS-CoV-2 virus that damaged endothelial cell membranes. The second was that of extensive vascular thrombosis with microangiopathy and occlusion of alveolar capillaries. The third feature was the observation of significant angiogenesis in the lungs of COVID-19 patients. The first finding of the presence of the SARS-CoV-2 virus within the endothelial cell is suggestive of the direct viral effects. This, accompanied by the perivascular inflammation may be contributing factors responsible for the endothelial dysfunction associated with COVID-19 (Ackermann et al., 2020). Furthermore, there is evidence indicating complement dysregulation associated with thrombotic microangiopathy as one of the most prominent pathophysiological mechanisms in addition to or as part of thrombo-inflammation in COVID-19 (Wang et al., 2021).

### Involvement of the Complement System in COVID-19

Patients presenting with severe COVID-19 have several abnormal parameters on their hematology profiles, such as increased level of D-dimer, thrombocytopenia, decreased fibrinogen levels, and prolonged prothrombin time (Tang et al., 2020). Another theory to understand the pathophysiology of thrombosis in COVID-19 patients is the indirect activation of endothelial cells which are mediated by complement. When the body is threatened by an infection, platelets, coagulation factors, and innate immune effector systems interact to form clots in a process termed immunothrombosis (Henry et al., 2020). While this is a protective mechanism, uncontrolled and widespread immunothrombosis may result in potentially serious microangiopathy with catastrophic consequences such as general or COVID-19 induced adult respiratory distress syndrome (Henry et al., 2020) and (Frantzeskaki et al., 2017).

Red blood cells are cells that express complement receptors and are capable of binding immune complexes through specific receptors. The study by Lam et al., 2021 reported on the deposition of complement (C3b and C4d) on circulating RBCs from hospitalized COVID-19 patients using flow cytometry (Lam et al., 2021). Evidence of the deposition of immune complexes and complement on RBCs may alter the rheology of RBCs and this promotes intravascular stasis and thrombosis that is key in the pathogenesis of virus associated lung injury (Lam et al., 2021). As mentioned previously, PS exposure on the surface of cells exposed to plasma results in thrombin generation and complement system activation. Phosphatidylserine-binding domains are present on coagulation proteins found in blood and this facilitates them to cluster together on exposed PS, resulting in an accelerated reaction and subsequent generation of thrombin. The exposed PS can lead to the activation of the complement system as well. Thus, both the coagulation and complement systems are integral role players in the complex clinical phenotype of thrombo-inflammation (Lind, 2021).

## Conclusion

Despite the fact that important physiological systems of coagulation and inflammation are viewed as separate entities, even discussed as separate chapters in physiology textbooks, the complications of COVID-19 illustrate how highly integrated and finely balanced these biological systems are with widespread crosstalk, all in preparation for any form of pending bodily injury. The latest scientific advancements achieved to treat and prevent COVID-19 so promptly, has been remarkable. However, the COVID-19 pandemic continues to wreak havoc as global death estimates approximates 5,5 million people (WHO COVID-19, 2022).

Complications seen in COVID-19 are as a result of a thrombotic process and can be defined as immuno-thrombo-inflammation as supported by current evidence (Lippi et al., 2021). This review also adds to research advocating the exploration of novel therapeutics such as drugs to amend PS exposure (Lind 2021), complement inhibitors (Lam et al., 2021), and drugs that can target the endothelium so as to treat or prevent vascular complications. It should be clear that basic biomedical and clinical medicine has to be combined if we are to achieve improved outcomes in COVID-19. The interplay between several biochemical, biophysical mechanisms in the pathogenesis of complications of COVID-19 is also a warning that to improve outcomes in severe COVID-19, a personalized therapeutic approach is likely needed for each individual based on personal risk factors profile and clinical presentation (Lippi et al., 2021). The crosstalk between eryptosis, endothelial dysfunction, coagulation and thrombosis in COVID-19, has been highlighted in this paper. The role of the RBC is clearly beyond that of oxygen transport. The study indicating the presence of complement activation products and viral antigens on RBCs in patients with COVID-19, is evidence that the RBC has been overlooked to achieve precision medicine which will eventually aim to improve outcomes in COVID-19 (Lam et al., 2021).
